# Absence of Medical Record Documentation of Advance Care Planning Status in At-Risk Emergency Department Patients

**DOI:** 10.7759/cureus.89248

**Published:** 2025-08-02

**Authors:** Jessica K Eygnor, Jennifer E Allen, William G Carmichael, Katie M Best, David B Burmeister, Katelyn L McLain, Zhe Chen, Justin M Stauffer, Courtney M Moore, Anthony Schinelli, David M Richardson, Matthew M McCambridge, Marna R Greenberg, Timothy J Friel

**Affiliations:** 1 Emergency and Hospital Medicine, Lehigh Valley Health Network/University of South Florida (USF) Morsani College of Medicine, Allentown, USA; 2 Optimizing Advanced Complex Illness Support (OACIS) / Palliative Medicine, Lehigh Valley Health Network/University of South Florida (USF) Morsani College of Medicine, Allentown, USA; 3 Quality and Patient Safety, Lehigh Valley Health Network/University of South Florida (USF) Morsani College of Medicine, Allentown, USA; 4 Medicine, Lehigh Valley Health Network/University of South Florida (USF) Morsani College of Medicine, Allentown, USA

**Keywords:** advance care planning, electronic medical record (emr), emergency department, goals of care, palliative care

## Abstract

Background

The majority of those aged 65 and older will visit the emergency department (ED) in the last six months of life. Knowing a patient’s goals of care is important, and existing medical records do not always represent them well. We set out to determine the baseline availability of advance directives and goals of treatment in those ED patients at increased risk for mortality.

Methods

This prospective cohort study included a sample of adult ED patients who had a mortality predictor by an End-of-Life (EOL) Deterioration Index-guided electronic best practice advisory (BPA) or admission to any of the network's intensive care units (ICU). Electronic medical record (EMR) abstraction was used to evaluate for documentation of healthcare proxy, healthcare power of attorney (POA), living will, advance care plans, or physician orders for life-sustaining treatment (POLST).

Results

A total of 9,321 patient encounters, representing 7,204 unique patients, were included in the analyzed sample. Most patients’ charts lacked advance care planning documentation such as healthcare proxy (98.7%, N=9200), healthcare POA (93.0%, N=8665), living will (94.6%, N=8816), advance care planning status (66.8%, N=6226), and POLST (95.8%, N=8928). Overall, urban sites had a larger percentage of encounters in which a high-risk patient might benefit from advance care planning discussions than rural sites. Females had a higher percentage of documentation across all variables of goals of care, with significant differences in healthcare POA (p < 0.001), advance directives (p < 0.001), and POLST (p = 0.008).

Conclusions

The majority of patients with a higher risk for mortality, as indicated by an EOL Deterioration Index-guided BPA or hospital ICU admission, do not have documentation in the EMR across all variables of goals of care.

## Introduction

Even before the COVID-19 pandemic, high-volume surges in the emergency department (ED) challenged clinical coverage for emergency patients [1]. The pandemic uncovered deficiencies in providing specialty-level palliative care in the ED's fast-paced, high-pressure milieu [2]. The ED is a common site for encountering older adults with advanced illness; 75% of those over 65 years old visit the ED in the last six months of life [3]. Therefore, it is a critical site for reviewing care goals [1]. Decisions made and treatments initiated in the ED can significantly impact the direction and quality of subsequent care [4]. Thus, it is essential for a patient’s documented electronic medical records (EMR) to accurately reflect their goals of care in the ED and beyond.

The need for readily available advance care planning status medical record documentation is expected to increase as the number of those aged 65 and older is expected to double by 2050 [5]. It should also be noted that older adults with advanced diseases often have individual and varying priorities when considering end-of-life (EOL) care [6]. Due to the increasing prevalence of this complex topic, we set out to determine the baseline availability of advance care directives and treatment goals in ED patients at advanced risk for mortality.

Data from this manuscript were presented, in part, at the Pennsylvania College of Emergency Physicians (PACEP) 2024 Scientific Assembly in Pocono Manor, PA (May 2-4, 2024); the Society for Academic Emergency Medicine (SAEM) 2024 Annual Meeting in Phoenix, AZ (May 14-17, 2024); the American College of Emergency Physicians 2024 Scientific Assembly in Las Vegas, NV (September 29-October 2, 2024); the PACEP 2025 Scientific Assembly in Pocono Manor, PA (April 24-26, 2025); and the SAEM 2025 Annual Meeting in Philadelphia, PA (May 13-16, 2025).

## Materials and methods

Setting and timeframe

This study was performed at a large northeast Pennsylvania hospital network comprised of nine EDs with an annual aggregate census of 313,555. Hospital sites included five nonurban settings and four urban settings, one of which was a level one trauma center. After Institutional Review Board approval, this prospective cohort study was performed during the six months from May 18 to November 18, 2023. During this time period, all advanced mortality risk ED patients were included in the study sample.

Inclusion and exclusion criteria

ED patients were included in the study if they were considered at advanced risk for mortality, defined by having mortality predicted by an EOL Deterioration Index-guided electronic best practice advisory (BPA). Epic’s EOL Index Cognitive Computing Model uses a penalized logistic regression to predict one-year mortality [7]. The model uses linear predictor inputs like patient demographics, comorbidities (examples such as cancer, diabetes, renal failure, and heart failure), labs, and medications with an associated weighted coefficient. These inputs are totaled to calculate a linear predictor (LP), which is then used in the standard logistic equation to compute an EOL Deterioration Index score [7]:

$\displaystyle \frac{1}{1 + e^{-LP}}$

Any patient with a total EOL index score of 30 or greater using this model automatically created a BPA and was included in the sample. The study also included patients admitted to intensive care units (ICU) (medical, surgical, cardiac, neurological, or trauma) to account for patients with acute deterioration not reflected in the model. Patients were excluded from the study if they were under the age of 18.

Data extraction and analysis

The study team used EMR abstraction to evaluate if there were documentation or records of the following: healthcare proxy, healthcare power of attorney, living will, advance care plans, or physician orders for life-sustaining treatment (POLST). A POLST form is a portable medical order summarizing a patient’s wishes for EOL care [8]. This electronic chart abstraction was verified for accuracy through a series of two manual chart audits. This involved study team members taking random samples from the cohort and comparing the variables in the automated extractions to the actual medical record to ensure that the abstraction process was correct. Minor changes were made in the electronic abstraction process to account for discrepancies found. For instance, the presence of a POLST or a living will document was initially erroneously electronically counted as ‘present’ if a box was checked by the registrar or staff member that they had inquired if these documents existed, and not that they were present. On the revision, they were considered ‘present’ only if the POLST or living will documents existed and were in the record at the time of the encounter. After confirming that these coding abstraction definitions were corrected in the second manual audit, a cohort analysis was performed to calculate the percentage of patients missing each medical record document. Demographic data for the high-mortality-risk patients were also recorded, analyzed, and reported. Additionally, the number of encounters occurring at urban vs. nonurban sites was identified. Frequencies and percentages were used to report findings and comparisons. The chi-square test of independence was used to determine significant differences in documentation between sexes (α=0.05).

## Results

Over the six months of the study, 9,321 patient encounters, representing 7,204 unique patients, were included in the study sample (Table 1). The mean patient age was 72. 53.8% (N=3875) of the patients were male, and 46.2% (N=3329) were female. The mean EOL index (high risk ≥30) for the cohort was 37.6. Within this cohort, we identified that the vast majority of patients’ charts lacked documented advance care planning; 98.7% (N=9200) did not identify a healthcare proxy, 93.0% (N=8665) did not identify a healthcare power of attorney, 94.6% (N=8816) did not have a living will document on file, 66.8% (N=6226) lacked a documented advance care planning status, and 95.8% (N=8928) did not have a POLST present. 

**Table 1:** Summary of the “At Risk” Patient Cohort SD: Standard Deviation; EOL: End-of-Life; POA: Power of Attorney; ACP: Advance Care Planning; POLST: Physician Orders for Life-Sustaining Treatment

Variable	Value
Total Encounters (N)	9321
Seen at Urban or Suburban Sites, N (%)	6215 (66.7)
Seen at Rural Sites, N (%)	3106 (33.3)
Total Individual Patients (N)	7204
Male, N (%)	3875 (53.8)
Female, N (%)	3329 (46.2)
Age (mean ± SD)	72 ± 15.9
EOL Index (mean ± SD; high risk ≥30)	37.6 ± 358.3
Absent Documentation (By Encounter)	
Healthcare Proxy Not Identified, N (%)	9200 (98.7)
Healthcare POA Not Identified, N (%)	8665 (93.0)
Living Will Documents Not on File, N (%)	8816 (94.6)
ACP Status Not Documented, N (%)	6226 (66.8)
POLST Not Present, N (%)	8928 (95.8)

Overall, urban sites had a larger percentage of encounters in which a high-risk patient might benefit from advance care planning discussions than rural sites (Tables 2, 3). Of the encounters in this high-risk cohort, 66.7% (N=6215) occurred at urban hospital sites, comprising 7.5% of the six-month average census for those four hospitals (N=83168). In contrast, 33.3% (N=3106) of encounters occurred at nonurban sites, comprising only 4.5% of the six-month average census for those five hospitals (N=69584). Urban sites had a similar percentage of encounters with male (54.7%) and female (45.3%) high-risk patients compared to rural sites (53.1% male and 46.9% female).

**Table 2:** High-Risk Encounters at Urban and Rural Emergency Departments *Site A is a Level 1 Trauma Center; ED: Emergency Department

Type of Site	Hospital Site	Six-Month Average ED Census, N	High-Risk Encounters, N (%)
Urban	A*	37,507	3,975 (10.6%)
	B	23,720	1,792 (7.6%)
	C	12,036	32 (0.3%)
	D	9,905	416 (4.2%)
	Urban Site Subtotals	83,168	6,215 (7.5%)
Rural	E	14,407	900 (6.2%)
	F	17,248	908 (5.3%)
	G	11,039	153 (1.4%)
	H	20,047	1,006 (5.0%)
	I	6,844	139 (2.0%)
	Rural Site Subtotals	69,585	3,106 (4.5%)
All	Totals	152,753	9,321 (6.1%)

**Table 3:** High-Risk Encounters at Urban and Rural Emergency Departments by Sex *Site A is a Level 1 Trauma Center.

Type of Site	Hospital Site	High-Risk Encounters, N (%)	Females, N (% by Site)	Males, N (% by Site)
Urban	A*	3,975 (42.6%)	1,772 (44.6%)	2,203 (55.4%)
	B	1,792 (19.2%)	835 (46.6%)	957 (53.4%)
	C	32 (0.3%)	15 (46.9%)	17 (53.1%)
	D	416 (4.5%)	193 (46.4%)	223 (53.6%)
	Urban Site Subtotals	6,215 (66.7%)	2,815 (45.3%)	3,400 (54.7%)
Rural	E	900 (9.7%)	458 (50.9%)	442 (49.1%)
	F	908 (9.7%)	456 (50.2%)	452 (49.8%)
	G	153 (1.6%)	77 (50.3%)	76 (49.7%)
	H	1,006 (10.8%)	416 (41.4%)	590 (58.6%)
	I	139 (1.5%)	51 (36.7%)	88 (63.3%)
	Rural Site Subtotals	3,106 (33.3%)	1,458 (46.9%)	1,648 (53.1%)
All	Totals	9,321 (100%)	4,273 (45.8%)	5,048 (54.2%)

In regard to sex specific outcomes, males had an average age of 70 years and females had an average age of 73 years. The mean EOL index (high risk ≥30) was 37 for females and 38 for males. High-risk factors (EOL Index and ICU stay) were similar between males and females. Females had a higher percentage of documentation in the EMR across all variables of goals of care; the majority of these differences met statistical significance (Table 4).

**Table 4:** Advance Care Planning Documentation and High-Risk Factors by Sex *Significance: p <0.05, Determined by the Chi-square Test of Independence POA: Power of Attorney; ACP: Advance Care Planning; POLST: Physician Orders for Life-Sustaining Treatment; EOL: End-of-Life; ICU: Intensive Care Unit

Variable	Males (n, %) by Encounter N= 5048	Females (n, %) by Encounter N= 4273	P- value
Documentation on File			
Healthcare POA Listed	314, 6.2%	342, 8.0%	<0.001*
Living Will Documents Present	258, 5.1%	247, 5.8 %	0.155
Advance Directive Present	1285, 25.5%	1244, 29.1%	<0.001*
ACP Note Present	437, 8.7 %	411, 9.6%	0.108
POLST Present	187, 3.7%	206, 4.8%	0.008*
High Risk Factors			
High EOL Index (≥ 30)	2183, 43.2%	1892, 44.3%	0.316
ICU Stay Documented	2398, 47.5%	2047, 47.9%	0.069

## Discussion

Since the 1990 passing of the Patient Self-Determination Act, hospitals nationwide have grappled with how and when to discuss end-of-life care with patients and inform them of their legal right to document their medical preferences [9]. Three decades later, health systems are still struggling with this question. Some might argue that the ED is not the correct place for advance care planning discussions due to the constrained time to develop a patient-clinician relationship. However, others argue that an ED visit can signal clinical decline and present a significant opportunity to introduce conversations about preferences in care [1, 10].

Previous work has shown there is a lack of advance care planning documentation among ED patients in general [11]. However, the sample created in this study was selective for individuals who would reap the highest benefits from advance care planning via inclusion if they had a mortality predictor by an EOL Index-guided BPA or ICU admission as previously described. Thus, the results of this study showing that there is a severe lack of advance care planning documentation among the most high-risk mortality individuals in the ED are particularly concerning. Additionally, these data could reflect the presence of a healthcare-wide care gap for advanced-risk patients that extends beyond the ED.

A greater percentage of encounters from our high-risk patient cohort occurred at urban rather than nonurban hospitals. This is not attributable to larger patient volume at urban sites, since high-risk patient encounters also constituted a larger proportion of the collective ED census of the urban sites compared to the rural sites. This may reflect a tendency for patients with more severe conditions to be treated in urban settings over rural ones, especially since the urban hospitals in this study included a level one trauma center. The lack of advance care planning documentation for these high-risk patients, regardless of treatment site, demonstrates a need for both rural and urban hospitals to better prioritize early determination of patients’ goals of care.

While there was a general paucity of documentation across our entire study cohort, female patients had more documentation than male patients across all variables and were significantly more likely to have healthcare power of attorney, advance directives, and POLST documented in their EMR. It is unlikely that these disparities could be attributed to differences in age or severity of illness between the two groups, since males and females had similar average ages and high-risk factors in the data we examined. Future research is needed to determine the potential causes of these sex-specific differences and how to best address them in the clinical setting.

These results, along with prior data demonstrating that the need for advance care planning is expected to grow with an aging population in the coming years [5], highlight a need for increased specialty-level palliative care in the ED. Since the ED is often the first line of defense for patients at advanced risk for mortality [3], the implementation of more regulated care documentation in this department would likely have a positive impact on documentation practices throughout the medical community. Resources exist for integrating palliative care services in the ED [12], but further research is needed on the most effective methods, especially for increasing advance care planning documentation. One study found an improvement in palliative consultations and hospice referrals after the introduction of streamlined workflows and the use of simulations to educate ED staff [13]. Despite differing opinions on which department is best to integrate these practices, advance care planning initiatives taken by the ED should be welcomed as a positive change, considering the apparent need for more organized documentation of patient end-of-life care goals underscored in this study.

Limitations

A limitation of this study was its geographical restriction to Northeast Pennsylvania. Lehigh County, Northampton County, and Pennsylvania, in general, have a higher percentage of White residents than the United States; therefore, many minority groups are likely underrepresented in this study [14]. However, research suggests that advance care planning documentation is even more sparse among minority populations. A California study demonstrated that White adults are 50% more likely to have advance directives than African American adults [15]. Another study in California showed that non-Hispanic White adults are much more likely to report that they have even heard of palliative care than Hispanic adults or non-Hispanic Black adults [16]. Therefore, the profound results of this study, conducted in a region with a relatively low minority population, may be amplified across the more diverse population of the United States.

Our study did not provide a multivariate analysis of outcomes primarily due to the very low documentation rate of the majority of variables evaluated. Additionally, the six-month study period was somewhat arbitrarily determined by the study team. The impact of the convenience sample of the exact months of the year chosen or the length of the study period on the potential bias it caused on study outcomes is not actually known. It is unlikely that a different time of year or a longer period of study would have had profound differences in the overall abysmal rates of documentation found.

Another limitation of this study may be the use of electronic chart abstraction. Family members or patients may have physical copies of advance care documents during their visit that were not counted in this study, leading to underreporting of documentation. However, since it is preferable for hospital systems to have goals of care documentation in the EMR at the time of care, the lack of documentation reported in this study still demonstrates an important gap in recording patients’ goals of care. Future research should also consider how to ensure all advance planning documentation is recorded in the medical record.

## Conclusions

In our study, the majority of patients who had a higher risk for mortality, as indicated by an EOL Index-guided BPA or hospital ICU admission, did not have a healthcare proxy or power of attorney identified, a living will present, advance directives documented, or a POLST in the EMR. Future investigation is still needed to understand the causes underlying this lack of documentation in the ED patient EMR. Altogether, this study highlights the need for future research into an evidence-based ED-initiated intervention to close the gap in advance care directive documentation for at-risk patients. In particular, there appear to be some sex-specific differences that may indicate a need for sex-specific intervention development. Overall, the findings of this study demonstrate an important opportunity for future supportive care discussions and the determination of ED evidence-based interventions to close the existing gap.
